# Effect of Sugar Beet Genotype, Planting and Harvesting Dates and Their Interaction on Sugar Yield

**DOI:** 10.3389/fpls.2018.01041

**Published:** 2018-07-18

**Authors:** Zivko Curcic, Mihajlo Ciric, Nevena Nagl, Ksenija Taski-Ajdukovic

**Affiliations:** Institute of Field and Vegetable Crops, Novi Sad, Serbia

**Keywords:** planting date, harvest date, environment, interaction, AMMI, genotype, sugar yield

## Abstract

Climate changes are affecting the plant production, including sugar beet growing especially in the southern and central parts of the Europe. Modifying the sowing and harvesting dates are one of the most often used adaptations in sugar beet cultivation. The aim of this study was to assess the interactions between planting date and sugar beet genotypes for different harvest dates with recommendation for duration of vegetation period for specific hybrids in order to achieve the best performance and to evaluate influence of climatic factors on sugar yield. Three-way analysis of variance and AMMI (Additive main effect and multiple interactions) analysis were performed to investigate interaction between main factors. Analysis of variance revealed that genotypes (G), planting date (PD), harvest date (HD) and interaction G × PD significantly affected sugar yield in 2016. In 2017 genotypes, planting date, harvest date and G x PD interaction significantly affected sugar yield on probability level of 1%, while PD × HD interaction had significant effect on probability level of 5%. Results of AMMI analysis enabled discrimination of genotypes with the highest level of stability in certain planting dates. Hybrids with combined yield and sugar content (NZ type) should have the advantage in earlier planting dates compared to of sugar beet hybrids with higher sugar content (Z type). However, in shortened vegetation period Z type hybrids are more stable and with better sugar yield results. Results of our study suggest that delaying the harvest date decreases differences between sugar yields obtained from hybrids sown in different planting dates. Major factors in the study affecting sugar yield were growing degree days, insolation and number of days from planting to harvest.

## Introduction

Trends of high average temperatures, with increased frequency of droughts, are affecting plant production throughout the Europe, but southern and central parts of the continent are especially endangered (Schär et al., 2004; Spinoni et al., 2015). Temperate regions of Pannonian plain and countries such as Hungary, Serbia, Croatia, and Romania are likely to be strongly affected by climate changes followed by summer heat waves and droughts during the vegetation, without possibilities for effectively shifting crop cultivation to other parts of the year (Olesen et al., 2011). A wide range of adaptations in agricultural practice (irrigation, intercropping, mineral nutrition etc.) are used in many European regions to minimize the negative impacts of climate change on crop production. According to White et al. (2011) adjusting the sowing date is by far the most frequently investigated climate change adaptation option. Yield potential of many crops is highly influenced by sowing date since it determines the length of vegetation period and the amount of captured radiation (Van Ittersum and Rabbinge, 1997).

Plant growth, development and, finally, yield are the result of genetic composition, the environmental effects and the interaction of these two factors. Phenomenon of the genotype by environment interaction (GEI) is always present in the crop production causing genotypes to have different results and ranks in various environmental conditions (Ndhlela et al., 2014). Environments differ in the amount and quality of inputs and stimuli that they convey to plants including, e.g., the amount of water, nutrients or incoming radiation (Malosetti et al., 2013). Often GEI is associated and explained with genetically terms of adaptation and stability (Dimitrijević and Petrović, 2000; Das et al., 2010). Various statistical methods such as regression analysis, nonparametric statistics and multivariate models are used for investigation and interpretation of this phenomenon and evaluation of different genotypes (Gauch et al., 2008). Additive main effect and multiple interactions (AMMI) is one of the most used methods for interpretation of GEI data. AMMI associates the analysis of variance (ANOVA) with principal component analysis (PCA) in one method (Gauch, 2013). In final phase AMMI removes the additive effect from interaction by ANOVA and then analyses interaction structure using PCA method.

Sugar beet is the main sugar producing crop in the Europe, and since it has been grown in the wide range of environmental conditions, successful management and production of the crop often represent a challenge for breeders and farmers (Jaggard et al., 2007; Hergert, 2010). Choosing the sugar beet hybrid with high yield potential is important as well as good adapted agronomic measures and practices, synchronized with requirements and needs of the plant (ulaković et al., 2015). Commercially, the sugar beets most important trait is sugar yield (Bosemark, 2006), which is strongly influenced by environment and highly correlated to root yield and sugar content (Powers et al., 1963; Schneider et al., 2002; Hoffmann et al., 2009).

Various types of sugar beet hybrids, developed by many seed companies, are present in the southern and central parts of the Europe do not have the same requirements and reactions to the local environmental effects. In the Serbia are usually grown two types of hybrids: Z type, with high levels of sugar content, intended for early harvest; and NZ type, with balanced root yield and sugar content, designed for medium and late harvest (Ludecke, 1953; Bosemark, 2006). Sowing period of sugar beet in the Serbia starts in the middle of March ends in April and might last for 45 days. During recent years campaigns of harvesting and processing beet roots were often prolonged from the end of August to the beginning of December and lasted for approximately 120 days.

Considering the long period from sugar beet sowing to harvest, the aim of this study was to: (i) detect the interactions between planting date and hybrids for two harvest dates; (ii) recommend sugar beet hybrids with the best performance for the specific vegetation period as useful tool for increasing the sugar yield; and (iii) to determine the effect of environmental variables on sugar yield.

## Materials and methods

### Plant material

The hybrids included in the study have been selected in order to obtain a high diversity regarding yield and quality properties. The sugar beet hybrids chosen for the first year of study were (i) newly registered hybrids with the best results from 2016 registration trials organized by the Ministry of Agriculture, Republic of Serbia (Tesla, Grandiosa, Beetle) and (ii) hybrids with high market share (Tibor, Tajfun). Since newly registered hybrids were not introduced on larger acreages, in second year of the field trials were tested hybrids registered in the last five years, with high market share in 2017 (Koala, Eduarda, Leopolda, Vandana). The hybrids were developed by different seed companies and belonged to Z and NZ type (Table 1).

**Table 1:** Sugar beet hybrids used in trials.

**Hybrid**	**Company**	Type of hybrid^*^	**Year of registration**	**Harvest time recommendation**
Tibor	Strube	Z	2004	Early/medium
Tajfun	Maribo	Z	2008	Early/medium
Tesla	Strube	Z	2016	Early/medium
Beetle	SES van der Have	NZ	2016	Medium/late
Koala	SES van der Have	Z	2013	Early/medium
Grandiosa KWS	KWS	NZ	2016	Medium
Eduarda KWS	KWS	NZ	2014	Harvest flex
Leopolda KWS	KWS	ZN	2014	Early/medium
Vandana KWS	KWS	NZ	2016	Medium

* *Z type, hybrid with high levels of sugar content; NZ type, hybrid with balanced root yield and sugar content*.

### Field trials

The field trials were carried out at the fields of Institute of field and vegetable crops, Novi Sad (IFVCNS), at the location Rimski Šančevi (45°20′N, 19°51′E) during two successive years (2016 and 2017). Experiment was organized in the randomized complete block design (RCBD) with four replications. Basic plot size was 20 m^2^, with four rows 10 m long and row spacing 0.5 m. Soil type was chernozem with characteristics presented in the Table 2. Sowing was performed by seed drills on four different planting dates (PD) (Table 3) with the distance of 0.09 m in row and 0.5 m between the rows. After the development of the second pair of leaves, the seedlings were singled out to a final, recommended crop density of 100,000 plants/ha. Standard agricultural practices for sugar beet growing were applied during the vegetation period. Roots were harvested manually on two harvest dates (HD) (Table 3). Combinations of different planting and harvest dates were considered as different trial environments (Table 3). The root yield (RY) was determined by measuring the weight of roots from two middle rows and recalculating it as t/ha. Root samples were analyzed in the Laboratory for sugar beet root quality testing of at IFVCNS. Sugar content (SC) was measured according to polarimetric method. Sugar yield (SY) was calculated following the equitation: SY = RY × SC.

**Table 2:** Soil characteristics in 2016 and 2017.

**Year**	**Humus (%)**	**pH**		**P_2_O_5_ (mg/100g)**	**K_2_O (mg/100g)**
		**H_2_O**	**nKCL**		
2016	2.57	7.23	8.17	30.6	30.9
2017	2.34	6.92	7.82	20.6	29.5

**Table 3:** Combinations of different planting and harvest dates named as trial environments.

**Planting date**	**Harvest date**		**Year**
	**13. September**	**28. October**	
18.03.	En1-1-16	En1-2-16	2016
25.03.	En2-1-16	En2-2-16	
31.03.	En3-1-16	En3-2-16	
13.04.	En4-1-16	En4-2-16	
	**22. September**	**7. November**	
25.03.	En1-1-17	En1-2-17	2017
01.04.	En2-1-17	En2-2-17	
10.04.	En3-1-17	En3-2-17	
18.04.	En4-1-17	En4-2-17	

### Environmental conditions

Data on daily maximum and minimum temperatures, rainfall and insolation were obtained from meteorological station located less than 1 km away from the experimental plots. The number of days (DNo) was calculated from planting to harvest date. Thermal time (growing degree-days, GDD) was calculated by summing the daily values of mean temperatures minus the threshold value of 3°C (Milford et al., 1985), from the planting to the harvest date. Weather conditions in the years 2016 and 2017 differed especially in the precipitation, average temperatures and insolation (Table 4, Supplementary Table 1). In 2016 the amount of rainfall and its distribution were close to the sugar beet monthly requirements (Vučić, 1992). In 2017 severe summer drought and high temperatures had large negative impact on sugar beet crop. In 2016, the first autumn frosts were recorded on October 6 for a period of 3 days, while the appearance of frost in 2017 was not recorded before the second harvest date (Republički hidrometeorološki zavod RepublikaSrbija, 2017).

**Table 4:** Summary of environmental variables for trial environments.

**Environment**	**DNo**	**GDD (^°^C)**	**Insolation (h)**	**Precipitation (mm)**	**Temperature (**^**°**^**C)**		
					**Tmn**	**Tmx**	**Tma**
En1-1-16	179	2,755.3	1,499.7	380.2	11.83	25.82	18.37
En2-1-16	172	2,725.0	1,461.4	354.4	12.19	26.37	18.82
En3-1-16	166	2,689.3	1,427.8	348.4	12.54	26.78	19.18
En4-1-16	153	2,524.2	1,323.3	346.2	12.87	27.14	19.48
En1-2-16	224	3,195.7	1,718.2	461.8	10.87	24.75	17.31
En2-2-16	217	3,165.4	1,679.9	436.0	11.14	25.15	17.63
En3-2-16	211	3,129.7	1,646.3	430.0	11.38	25.43	17.88
En4-2-16	198	2,964.6	1,541.8	427.8	11.56	25.62	18.02
En1-1-17	181	2,954.2	1,748.2	299.2	11.46	27.45	19.26
En2-1-17	175	2,902.7	1,692.0	299.2	11.83	27.82	19.62
En3-1-17	165	2,816.2	1,626.0	297.0	12.14	28.26	20.00
En4-1-17	157	2,743.1	1,566.0	293.6	12.54	28.69	20.41
En1-2-17	227	3,354.4	2,020.2	335.2	10.25	25.91	17.75
En2-2-17	221	3,302.9	1,964.0	335.2	10.51	26.15	17.98
En3-2-17	211	3,216.4	1,898.0	333.0	10.69	26.43	18.21
En4-2-17	203	3,143.3	1,838.0	329.6	10.94	26.68	18.45

*GDD, growing degree days; Tmn, minimum temperature; Tmx, maximum temperature; Tma, mean average temperature*.

### Data analysis

Factorial ANOVA for sugar yield data was computed using Statistica 13 software package (Dell Inc, 2015, StatSoft, Tulsa, OK, USA) and Duncan's multiple range tests for detection of statistically significant differences. Factors genotype, planting date, harvest date were assumed fixed. Values of *P* ≤ 0.05 were considered significant. GEI data were analyzed using computing environment (R Development Core Team, 2013). AMMI analysis was completed using Excel Biplot Macros (Lipkovich and Smith, 2002). Pearson correlation coefficients between environmental data and sugar yield were calculated. To identify the environmental variables discriminating between different lengths of growing season, principal component analysis (PCA) was performed on the correlation matrix, calculated from the mean values for each growing season (R Development Core Team, 2013).

## Results

In both years of research newly registered hybrids showed better performance compared to old hybrids (Tables 5A,B). The highest average sugar yield had hybrids Tesla, Grandiosa and Beetle in 2016, while best performing hybrids in 2017 were Eduarda, Koala and Vandana. Delayed harvest date increased sugar yield in 2016 and 2017. Regardless of the different HD, the third PD resulted in the highest sugar yield, while the latest PD had the lowest yield in first year of research. In 2017 the highest sugar yield was recorded for second HD, while the first HD had the lowest yield.

**Table 5:** Sugar yield of tested sugar beet hybrids in trial environments in 2016 (A) and 2017 (B).

**Environments**	**Sugar yield (t/ha)**					**Average**
	**Tibor**	**Tajfun**	**Tesla**	**Grandiosa**	**Beetle**	
**(A)**						
En1-1-16	8.89 ± 0.20	9.56 ± 0.05	9.96 ± 0.51	10.82 ± 0.78	9.49 ± 0.51	9.74^b^
En2-1-16	7.74 ± 0.32	9.00 ± 0.71	9.80 ± 0.14	8.67 ± 0.28	10.68 ± 0.91	9.18^b^
En3-1-16	10.2 ± 0.21	10.27 ± 0.28	10.61 ± 0.19	10.75 ± 0.41	10.42 ± 0.48	10.45^a^
En4-1-16	5.61 ± 0.37	5.12 ± 0.25	5.58 ± 0.38	4.49 ± 0.45	5.53 ± 0.26	5.27^c^
Average	8.11	8.49	8.99	8.68	9.03	8.66^b^
En1-2-16	9.64 ± 0.34	9.23 ± 0.40	10.97 ± 0.86	11.57 ± 0.75	11.27 ± 0.94	10.54^a^
En2-2-16	7.66 ± 0.48	8.56 ± 0.86	9.44 ± 0.47	9.82 ± 0.84	10.36 ± 0.52	9.17^b^
En3-2-16	9.85 ± 0.38	10.39 ± 0.48	11.06 ± 1.06	11.12 ± 0.67	10.95 ± 0.87	10.67^a^
En4-2-16	5.53 ± 0.33	4.97 ± 0.24	6.43 ± 0.49	5.64 ± 0.46	5.83 ± 0.24	5.68^c^
Average	8.17	8.28	9.47	9.54	9.60	9.01^a^
Mean	8.14^b^	8.39^b^	9.23^a^	9.11^a^	9.32^a^	
**Environments**	**Sugar yield (t/ha)**						**Average**
	**Tibor**	**Tajfun**	**Eduarda**	**Koala**	**Leopolda**	**Vandana**	
**(B)**							
En1-1-17	9.59 ± 0.46	9.01 ± 0.49	10.23 ± 0.71	10.05 ± 0.79	7.98 ± 0.56	10.21 ± 0.24	9.51^b^
En2-1-17	7.46 ± 0.32	8.36 ± 0.63	8.38 ± 0.18	8.40 ± 0.34	7.76 ± 0.57	7.73 ± 0.61	8.02^c^
En3-1-17	8.50 ± 0.24	6.87 ± 0.39	7.91 ± 0.71	9.01 ± 0.93	7.47 ± 0.52	7.94 ± 0.32	7.95^c^
En4-1-17	7.90 ± 0.22	8.19 ± 0.08	6.95 ± 0.22	8.05 ± 0.41	8.48 ± 0.48	7.75 ± 0.49	7.89^c^
Average	8.36	8.11	8.37	8.88	7.92	8.41	8.34^b^
En1-2-17	10.39 ± 0.88	10.01 ± 0.15	11.72 ± 0.20	11.87 ± 0.74	9.09 ± 0.49	12.66 ± 0.96	10.96^a^
En2-2-17	9.06 ± 0.73	9.41 ± 1.00	9.58 ± 0.58	9.94 ± 0.50	9.90 ± 0.68	9.40 ± 0.67	9.55^b^
En3-2-17	10.89 ± 0.38	10.10 ± 0.44	10.29 ± 0.62	10.44 ± 0.67	10.79 ± 0.32	10.04 ± 0.46	10.43^a^
En4-2-17	10.28 ± 0.74	9.84 ± 0.49	10.16 ± 0.51	11.76 ± 0.60	10.23 ± 0.39	10.45 ± 0.31	10.45^a^
Average	10.15	9.84	10.44	11.01	10.00	10.64	10.34^a^
Mean	9.26^ab^	8.97^b^	9.40^ab^	9.94^a^	8.96^b^	9.52^ab^	

*Values followed by the same letter(s) are not significantly different (Duncan's Multiple Range test at p < 0.05)*.

According to three-way factorial ANOVA genotypes, PD, HD and G x PD interaction significantly affected sugar yield in 2016 (Table 6). PD accounted for 88.22% of total sum squares, while genotypes and G × PD interaction accounted for 5.04 and 3.97%, respectively. In 2017 genotypes, PD, HD, and G × PD interaction affected sugar yield on probability level of 1%, while PD × HD interaction had effect on probability level of 5%. HD effects participated in total variance with 55.51%, PD 16.08%, G × PD interaction 13.54%, genotypes 6.28%, while PD × HD interaction accounted for 3.7% of total sum squares.

**Table 6:** Summary ANOVA for sugar yield in 2016 and 2017.

**Source of variation**	**2016**					**2017**				
	**SS**	**df**	**MS**	**F**	**% of SS**	**SS**	**df**	**MS**	**F**	**% of SS**
G	36.8	4	9.2	7.75**	5.04	21.84	5	4.37	3.58**	6.28
PD	643.9	3	214.66	180.90**	88.22	55.89	3	18.63	15.28**	16.08
HD	4.98	1	4.98	4.20*	0.68	192.9	1	192.9	158.2**	55.51
G × PD	28.9	12	2.41	2.03*	3.97	47.0	15	3.14	2.57**	13.54
G × HD	5.71	4	1.43	1.2	0.78	1.59	5	0.32	0.26	0.46
PD × HD	3.46	3	1.15	0.97	0.47	12.86	3	4.29	3.52*	3.7
G × PD × HD	6.06	12	0.5	0.43	0.83	15.37	15	1.02	0.84	4.42
Error	142.4	120	1.19			175.61	144	1.22		

SS, sum of squares; MS, mean squares; df, degrees of freedom; G, genotype; PD, planting date; HD, harvest date. *,** indicate the significance levels of P < 0.05 and P < 0.01.

The AMMI ANOVA (Table 7A) showed that in 2016 genotypes and PD had significant effects for both HD, but interactions were significant only for the first HD. In 2017 sugar yield in both HD were influenced by PD and G × PD interaction, while genotypes effects were significant only for second HD (Table 7B). Contribution of G x PD interaction varied from 3.89% in 2016, to 46.87% in 2017. PD had the greatest contribution to total variation in 2016. In 2017, PD had the greatest contribution to total variation in the first HD, while for the second HD the interaction G x PD contributed the most. Effect of genotype increased in second HD for both years.

**Table 7:** Additive main effect and multiplicative interaction (AMMI) analysis of variance (ANOVA) for sugar yield in 2016 (A) and 2017 (B).

**Source of variation**	**df**	**1st HD**				**2nd HD**			
		**SS**	**MS**	***F-*value**	**% of SS**	**SS**	**MS**	***F*-value**	**% of SS**
**(A)**									
Total	79	402	5.09			465.3	5.89		
Treatments	19	355.7	18.72	23.62**		369.2	19.43	14.17	
Genotypes	4	9.2	2.31	2.91*	2.59	33.3	8.32	6.07**	13.52
PD	3	323.1	107.69	156.72**	90.83	324.4	108.13	42.86**	82.59
Block	12	8.2	0.69	0.87		30.3	2.52	1.84	
G x PD	12	23.4	1.95	2.46*	6.58	11.6	0.96	0.70	3.89
IPCA 1	6	13.9	2.32	2.92*		8.1	1.35	0.99	
IPCA 2	4	9.5	2.38	3.00*		2.8	0.7	0.51	
Residuals	2	0	0.01	0.02		0.7	0.33	0.24	
Error	48	38.1	0.79			65.8	1.37		
**(B)**									
Total	95	151.33	1.593			178.88	1.883		
Treatments	23	79.91	3.474	3.5**		74.69	3.247	2.57**	
Genotypes	5	8.4	1.681	1.69	10.51	15.03	3.005	2.37*	20.12
PD	3	44.1	14.698	14.86**	55.19	24.66	8.22	3.49*	33.01
Block	12	11.87	0.989	1.00		28.26	2.355	1.86	
G x PD	15	27.41	1.827	1.84*	34.30	35.01	2.334	1.84*	46.87
IPCA 1	7	18.46	2.637	2.66*		29.57	4.224	3.34**	
IPCA 2	5	7.18	1.436	1.45		3.58	0.717	0.57	
Residuals	3	1.78	0.592	0.60		1.85	0.617	0.49	
Error	60	59.56	0.993			75.93	1.266		

IPCA, interaction principal component axis; HD, harvest date; PD, planting date; df, degrees of freedom; SS, sum of squares; MS, mean squares. *,** indicate the significance levels of P < 0.05 and P < 0.01.

**Figure 1:**
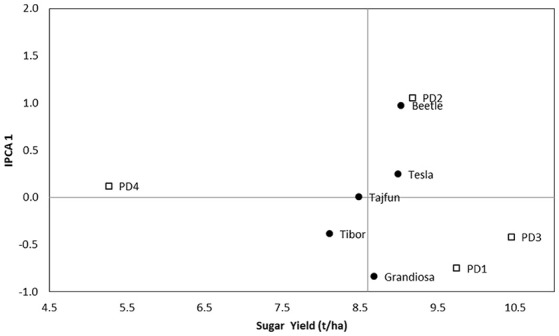
AMMI 1 biplot for sugar yield showing hybrids (black dots) and PD (squares) plotted against their IPCA1 scores in 2016 first HD.

In 2016 IPCA axes were not significant for the second HD, so AMMI biplots were made only for first HD. The AMMI 1 biplot indicate that hybrid Beetle had the best performance (9.03 t ha^−1^) while Tibor (8.11 t ha^−1^) had the lowest sugar yield among the PD (Figure 1). The most stable sugar beet genotypes in 2016 were Z type varieties Tajfun, Tesla and Tibor. The tested hybrids had the highest sugar yield on the third PD, while their lowest performance was on the fourth PD.

**Figure 2:**
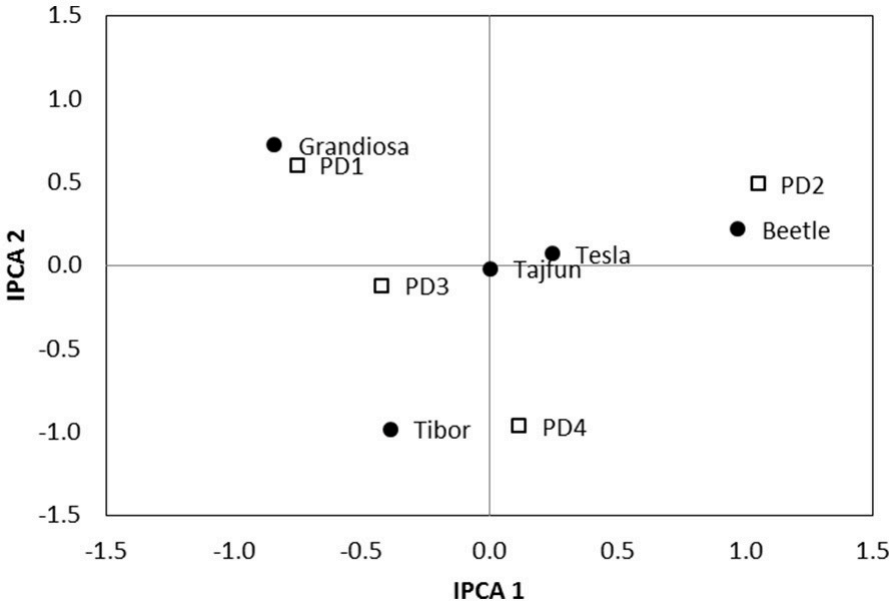
AMMI 2 biplot for sugar yield showing the interaction of IPCA2 against IPCA1 scores of five hybrids (black dots) across four PD (squares) in 2016.

AMMI 2 biplot indicated that certain hybrids had the potential for the best performance for the specific PDs (Figure 2). The hybrid Beetle showed the best performance in second PD, Grandiosa in the first PD, Tajfun and Tesla in the third PD. The close position of Tajfun and Tesla indicates that both hybrids would perform best in the similar environmental conditions.

**Figure 3:**
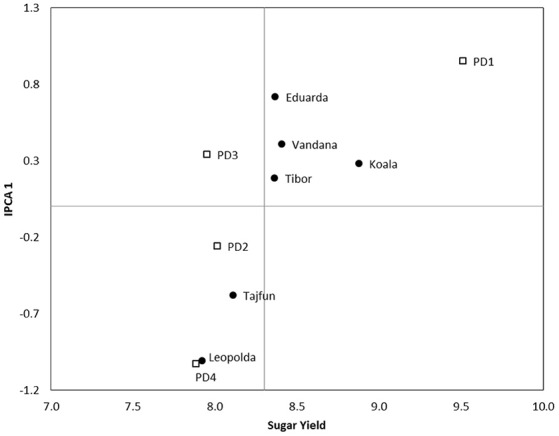
AMMI 1 biplot for sugar yield showing hybrids (black dots) and PD (squares) plotted against their IPCA1 scores in 2017 first HD.

According to the AMMI 1 biplot of the first HD in 2017 the best performance had Z type hybrid Koala (8.88 t ha^−1^), with small interaction score and relatively good level of stability, while the lowest sugar yield had Leopolda (7.92 t ha^−1^) (Figure 3). Beside Koala, stable sugar beet genotype for the first HD in 2017 was Z type hybrid Tibor. The tested hybrids had the highest performance on the first PD while the lowest results were recorded for fourth PD. Placement of both these planting dates indicated low level of stability for sugar yield. According to the positions of PD2 and PD3, although they were under-average environments they were more stable.

**Figure 4:**
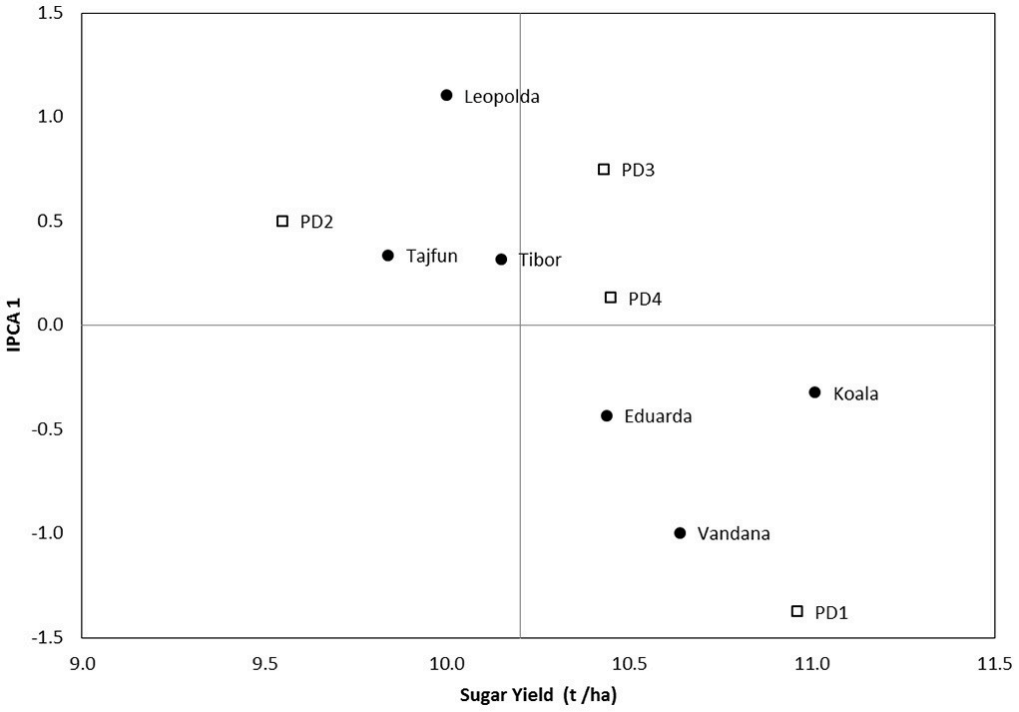
AMMI 1 biplot for sugar yield showing hybrids (black dots) and PD (squares) plotted against their IPCA1 scores in 2017 second HD.

In 2017, Koala was again the best performing hybrid (11.01 t ha^−1^) for the second HD, while the Tajfun had the lowest performance (9.84 t ha^−1^) (Figure 4). The highest stability showed Z type hybrids Tibor, Tajfun and Koala. The tested hybrids had the highest sugar yield on the first PD, while their lowest performance was on the second PD.

**Figure 5:**
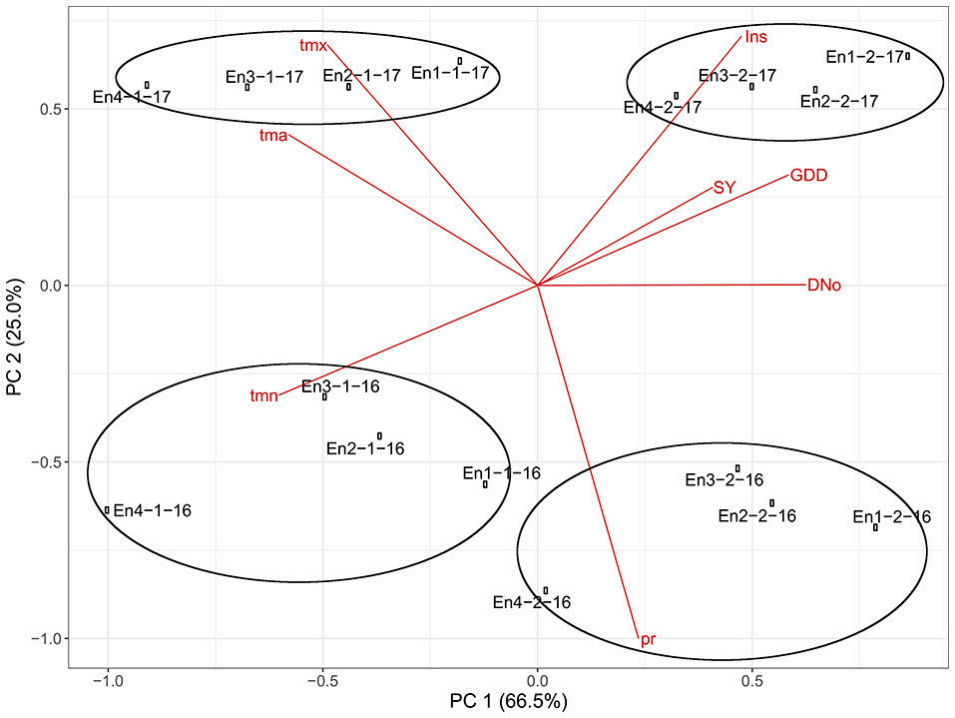
Plot of the principal component analysis (PCA) with eigenvectors for the environmental variables and eigenvalues for the environments in the trials. (DNo, number of days; GDD, growing degree days; Ins, insolation; pr, precipitation; tmn, average minimum temperature; tmx, average maximum temperature; tma, mean average temperature and SY, sugar yield).

To identify the combination of variables that better explained the environmental variation, we conducted principal component analysis (PCA) on the mean values of the environmental variables (Figure 5). The first two axes of the PCA accounted for 91.5% of the total variance, indicating that the most of the information held in the data could be summarized by projecting the points on the plain determined by these two axes. The first principal component (PC1) accounted for 66.5% of the expressed variation. PC1 was related to all environmental variables and sugar yield, with minimal effect of precipitation. Increases in PC1 were related to number of days, growing degree days, sugar yield and insolation. The negative direction of PC1 was related to minimum, maximum and mean average temperatures. The second principal component (PC2) accounted for 25% of the expressed variation. Increases in PC2 were related to insolation and average maximum temperature. The negative direction of PC2 was related to precipitation.

The points corresponding to the each environment were ploted in the Figure 5. The first group of environments in the upper right part of the figure, representing the 2017 second HD, indicates that they were characterized by large amount of insolation and GDD which agrees with the data shown in Table 4. The points corresponding to 2017 first HD were located in the left upper part of the plot and characterized with higher maximum average and daily average temperatures. The points belonging to the third and fourth groups were located in the lower part of the figure representing the environments of 2016 with higher minimal temperatures and larger precipitation which is in agreement with their meteorological background shown in Table 4.

**Figure 6:**
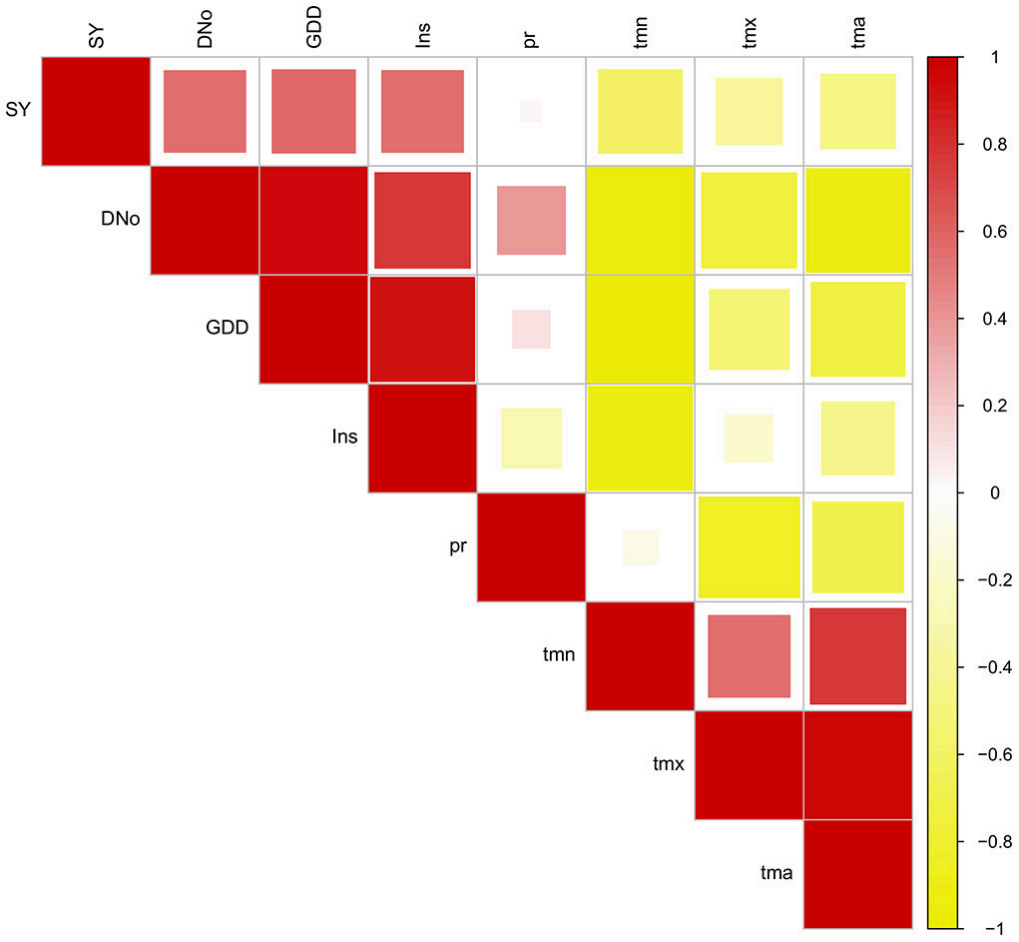
Pearson's correlation coefficients between environmental variables and sugar yield (DNo, number of days; GDD, growing degree days; Ins, insolation; pr, precipitation; tmn, average minimum temperature; tmx, average maximum temperature; tma, mean average temperature).

Correlations between environmental variables and sugar yield are represented in the Figure 6. Sugar yield was positively correlated with GDD (0.58), Ins (0.56), and DNo (0.56), while negative correlation was detected only with Tmn (−0.59). The cumulative variables, DNo, GDD, and Ins were positively correlated. Temperature variables were also positively correlated with each other, but were in the negative correlation with DNo, GDD and Ins. Precipitation was negative correlated with Tmx and Tma. These findings comply with the results of principal component analysis, presented in Figure 5.

## Discussion

In the study, performance of sugar beet hybrids through vegetation periods of different duration (different planting and harvest dates), were investigated, using the sugar yield as the main evaluation criterion. Previous investigations considering sugar beet cultivation (Jozefyová et al., 2003; Öztürk et al., 2008; Filipović et al., 2009; Hoffmann and Kluge-Severin, 2011; Bu et al., 2016) indicated that the earlier planting dates and later root harvest can be advantageous. Considering the difference in genetic potential, as well as the moment of technological maturity of sugar beet hybrids, our aim was to determine if there were interactions between the genotype and combination of different planting and harvesting dates.

For these purposes NZ and Z type sugar beet hybrids were tested in 16 different environments. In both years significant effect of the genotype, PD, HD, and G × PD interaction on sugar yield were recorded. Ratios between variances for these effects in the first year were similar to those obtained in study of Hoffmann et al. (2009), probably because the environmental conditions for sugar beet production were similar to those in Western Europe. In 2017 the ratio of the effects was quite different—the genotype effect remained similar, PD effect decreased, while effect of HD increased. Interaction G × PD increased and PD × HD became significant. In our opinion, probably because the changes in the variances of investigated effects are result of different environmental conditions in 2017, characterized with hot and dry summer, typical for the Pannonian plane. It is likely that increased genotype x environment interaction was mostly due to different reaction of tested hybrids to water deficit (Pidgeon et al., 2006) Similarly to previous studies of Wolf (1995), Bloch and Hoffmann (2005), and Ćurčić et al. (2012), there was no interaction between genotypes and HD, indicating that in autumn different sugar beet hybrids have very similar root development.

The ANOVA for the AMMI model showed that interaction G × PD was significant and twice as large as the genotype effect, which is in compliance with the research of Srivastava et al. (2008). Significant effect of G × E interaction in sugar beet field trials was recorded in many studies (Moradi et al., 2012; Hoberg et al., 2015; Al Jbawi et al., 2017). However, in the studies by Hoberg et al. (2016) and Shao et al. (2015), environment had predominant effect on sugar yield, while the effect of genotype x environment interaction had no significance. Campbell and Kern (1982) and Trimpler et al. (2017) concluded that among the numerous significant factors, year effect had the greatest influence on sugar beet production. In the studies of Sklenar et al. (2000) and Ćirić et al. (2017) G × E interaction was significant, but not the factor with the strongest effect on yield.

According to IPCA-1 biplots the genotypes and environments with high coordinates on IPCA-1 contributed to a greater extent to the G × E interaction while the genotypes and environments with IPCA-1 coordinates close to origin have little contribution in this interaction effect (Crossa et al., 1990). It could be concluded that in both years of research Z type hybrids were more stable and therefore less contributed to the interaction comparing to NZ type hybrids. The AMMI 2 biplot enabled connection of the specific genotypes and environments based on the G × E interaction scores. The grouping of the genotypes and the environments in the same quadrant indicated positive association between them. NZ type hybrids showed better adaptation to earlier PD, while Z type hybrids showed better reaction to third and fourth PD.

Although other factors such as soil condition could induce variability between environments, the results of the PCA showed that 91.5% of the environmental variation was explained by the environmental variables considered in the study. Since climate factors determine where and how plants grow, environmental variables (temperature, solar radiation, precipitation etc.) were used for description of environment as in Xu (2016). Weather conditions during the trial differed greatly. The first year had sufficient amount and good distribution of rainfall, while 2017 was characterized by extreme drought and exceptionally high temperatures in especially during July and August. Also, the absence of precipitation and lower temperatures in April 2017 resulted in lower number of plants per unit area.

The weather conditions between HD in tested years were different. Beside the frost appearance in 2016, the main difference was the insolation. In 2017 there was 50 h more of insolation between the HD than in 2016. This was probably one of the main reasons why sugar yield in 2017 increased by 2 t ha^−1^ between harvest dates, while in the same period in 2016, yield was increased only by 0.35 t ha^−1^. In the research of Kenter et al. (2006), there was positive correlation between root yield and solar radiation in the autumn, 175–200 days after planting.

To quantify influence of environmental variables on sugar beet hybrid performance, they were correlated to sugar yield. Although precipitation is often regarded as a major factor affecting sugar beet growth (Jaggard et al., 1998) in our study it was not significant for sugar yield, similarly to results of Kenter et al. (2006). There were positive correlations between GDD, Ins and DNo, which was in accordance to the research of Schnepel and Hoffmann (2016).

Considering the changing environmental conditions, as well as the introduction of new sugar beet hybrids in the production, research on the genotype and the planting date interaction for different harvest dates could provide the answer to the question which hybrids to grow under such conditions. The obtained results can help sugar factories to increase the total sugar yield per unit area, by recommending sugar beet hybrids for individual planting dates, with advanced planning of sugar beet harvest. Results of AMMI analysis in this study enabled discrimination of hybrids with the highest level of stability in certain planting dates. Priority for earlier planting dates should be given to NZ type of sugar beet hybrids. On the other hand, Z type sugar beet hybrids were more stable and achieved better results during shorter vegetation period. Our results suggest that by delaying the harvest, differences between sugar yield from different planting dates decrease and sugar yields from later harvesting dates are on the same level regardless of the planting date.

## Author contributions

ZC and MC designed and performed experiment, collected data, prepared the manuscript. NN and KT-A supervised the project, participated in preparation of manuscript.

### Conflict of interest statement

The authors declare that the research was conducted in the absence of any commercial or financial relationships that could be construed as a potential conflict of interest.
