# Supplementary material for: Effect of Sugar Beet Genotype, Planting and Harvesting Dates and Their Interaction on Sugar Yield
Source: Front Plant Sci. 2018 Jul 18;9:1041. doi: 10.3389/fpls.2018.01041 (PMC6058597; doi:10.3389/fpls.2018.01041)
Supplement: Supplementary file 1 [file Table_1.DOCX]

|  | Precipitation  (mm) | | Number of days with precipitation over 5mm | GDD  (°C) | Insolation (h) | Temperature (°C) | | | Number of day with max temperature over 30°C |
| --- | --- | --- | --- | --- | --- | --- | --- | --- | --- |
|  |  |  |  |  |  | Min | Max | Mean |  |
|  |  | 2016 | | | | | | |  |
| April | 75 | | 5 | 333.5 | 214.2 | 7.26 | 20.97 | 14.0 | 0 |
| May | 85 | | 3 | 423.9 | 237.9 | 10.41 | 23.37 | 15.8 | 5 |
| June | 143 | | 6 | 557.7 | 256.4 | 15.25 | 28.75 | 20.9 | 9 |
| July | 68 | | 2 | 605.6 | 326.4 | 15.51 | 30.76 | 21.9 | 20 |
| Avgust | 46 | | 4 | 545.2 | 292.7 | 13.98 | 29.02 | 20.5 | 13 |
| Septembar | 15 | | 1 | 188.7 | 79.8 | 13.16 | 29.53 | 20.4 | 6 |
| After first HD | 81 | | 4 | 455.1 | 221.3 | 7.30 | 20.76 | 12.6 | 6 |
|  |  | 2017 | | | | | | |  |
| April | 58 | | 2 | 252.9 | 191.0 | 4.67 | 18.21 | 11.1 | 0 |
| May | 83 | | 2 | 456.2 | 295.2 | 10.87 | 25.08 | 17.6 | 3 |
| June | 66 | | 1 | 592.1 | 350.1 | 14.60 | 31.07 | 22.9 | 20 |
| July | 12 | | 1 | 635.0 | 364.5 | 14.60 | 32.15 | 23.9 | 19 |
| Avgust | 18 | | 1 | 641.4 | 346.3 | 14.60 | 33.47 | 24.8 | 22 |
| Septembar | 61 | | 6 | 314.8 | 144.7 | 11.36 | 26.17 | 17.8 | 6 |
| After first HD | 35 | | 2 | 410.5 | 272.4 | 5.64 | 20.06 | 11.7 | 0 |

Supplement 1. Summary of environmental conditions in 2016 and 2017
